# Detection of Differential Levels of Proteins in the Urine of Patients with Endometrial Cancer: Analysis Using Two-Dimensional Gel Electrophoresis and *O*-Glycan Binding Lectin

**DOI:** 10.3390/ijms13089489

**Published:** 2012-07-27

**Authors:** Alan Kang-Wai Mu, Boon-Kiong Lim, Onn Haji Hashim, Adawiyah Suriza Shuib

**Affiliations:** 1 Institute of Biological Sciences, Faculty of Science, University of Malaya, Kuala Lumpur 50603, Malaysia; E-Mail: alanmukangwai@yahoo.com; 2 Department of Obstetrics and Gynaecology, Faculty of Medicine, University of Malaya, Kuala Lumpur 50603, Malaysia; E-Mail: limbk@ummc.edu.my; 3 Department of Molecular Medicine, Faculty of Medicine, University of Malaya, Kuala Lumpur 50603, Malaysia; E-Mail: onnhashim@um.edu.my; 4 University of Malaya Centre for Proteomics Research, Faculty of Medicine, University of Malaya, Kuala Lumpur 50603, Malaysia

**Keywords:** endometrial cancer, urine, biomarker, 2-dimensional gel electrophoresis, champedak galactose binding lectin, proteomics

## Abstract

Cancers can cause some proteins to be aberrantly excreted or released in the urine, which can be used as biomarkers. To screen for potential biomarkers for endometrial cancer (ECa), the urinary proteins from patients who were newly diagnosed with early stage ECa and untreated controls were separated using two-dimensional gel electrophoresis (2-DE) and followed by image analysis. The altered levels of zinc alpha-2 glycoprotein, alpha 1-acid glycoprotein, and CD59 were detected in the patients compared to the controls. In addition, the urine of the ECa patients was also found to contain relatively lower levels of a fragment of nebulin when the 2-DE separated urinary proteins were probed using champedak galactose binding (CGB) lectin. The different levels of the nebulin fragment were further validated by subjecting the urinary protein samples to CGB lectin affinity chromatography and analysis of the bound fractions by LC-MS/MS. Our data is suggestive of the potential use of the differentially expressed urinary proteins as biomarkers for ECa although this requires further extensive validation on clinically representative populations.

## 1. Introduction

Endometrial cancer (ECa) is one of the most common gynecological malignancies. It constitutes 6% of total cancers affecting women worldwide [1]. The diagnosis of ECa is very dependent on endometrial biopsy, which is invasive and highly subjective. As such, ECa is difficult to diagnose, particularly at the early stage of the malignancy. Up until now, no tumor marker has been established specifically for ECa, even though several serum markers that are known to be non-specific are currently being applied for its diagnosis [2]. Identification of proteins that are differentially expressed in the urine of ECa patients at an early stage may facilitate the development of a method to efficiently diagnose ECa non-invasively. These markers not only assist in the early and accurate identification of ECa patients, which is important for the patients’ survival, but also make the invasive biopsy procedure unnecessary for non-ECa patients.

Urine has become one of the attractive biofluids to screen for potential protein biomarkers. It promises a convenient method of disease detection and monitoring because it can be easily and noninvasively obtained from the patients. Urinary protein biomarkers have been reported to be used for the detection of cancers of the kidney, bladder and prostate [3]. One of the more recent and popular methods used to screen for the possible presence of potential biomarkers in the urine involves the proteomics technology [4,5]. When subjected to two-dimensional gel electrophoresis (2-DE), urinary proteins may be profiled on the basis of the differences in isoelectric point and molecular weight. These proteins may also be quantified by densitometry and identified using mass spectrometry and database searches. Since urine usually harbors hydrophilic molecules in the form of glycoproteins, lectins may be additionally employed in the proteomics method to specifically target the presence of proteins with specific oligosaccharide structures.

In the present study, the urine of patients who were newly diagnosed with ECa and control subjects was analyzed using 2-DE, densitometry and tandem mass spectrometry to screen for proteins that are differentially expressed. Aside from the conventional gel-based proteomics approach, a lectin that binds to the *O*-glycans of glycoproteins was also used to specifically profile the *O*-glycoproteins in the urine of the ECa patients and control subjects. Many *O*-glycoproteins had previously been implicated with cancer [6–9]. Hence, this lectin was chosen because it specifically binds to the oligosaccharide moieties of *O*-glycoproteins [10,11].

## 2. Results

### 2.1. Profiling of Urinary Proteins by 2-DE

**Figure 1:**
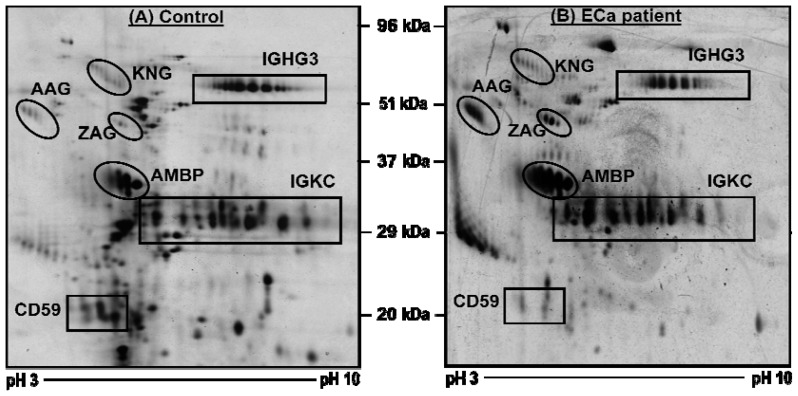
Typical two-dimensional gel electrophoresis (2-DE) urinary protein profiles. The labeled spot clusters are proteins which consistently appeared in profiles of control subjects. Panels (**A**) and (**B**) refer to the profiles of a control subject and a patient with stage IB endometrial cancer (ECa), respectively.

Comparable 2-DE urine protein profiles of patients with ECa and control subjects were obtained when their urinary protein samples were subjected to 2-DE followed by silver staining. Figure 1 shows representative urine proteome maps of the controls (panel A) and ECa patients (panel B). Seven protein clusters appeared to be consistently resolved in all the 2-DE maps of the patients and controls. When subjected to the MALDI-ToF MS/MS analysis, the protein clusters were identified as kininogen 1 (KNG), alpha-1 acid glycoprotein (AAG), zinc alpha-2 glycoprotein (ZAG), CD59, protein AMBP (AMBP), Ig gamma 3 chain C region (IgG3C) and Ig kappa chain C region (IgKC). Table 1 shows the mass spectrometric identification data of the urinary proteins that were resolved by 2-DE.

### 2.2. Image Analysis of 2-DE Separated Urinary Proteins

When image analysis was performed using the Image Master 2D Platinum Software version 7.0, it was apparent that the levels of the proteins detected in the urine of ECa patients were comparable to those of the controls, except for AAG, ZAG and CD59. The intensities of AAG and ZAG were more than 10-fold higher in the patients’ proteome map, whilst the CD59 protein cluster was barely detected in the 2-DE profiles of the patients’ urine samples. Table 2 demonstrates the average percentage of volume contribution of the seven detected urinary proteins.

### 2.3. Profiling of Urinary *O*-Glycoproteins

**Figure 2:**
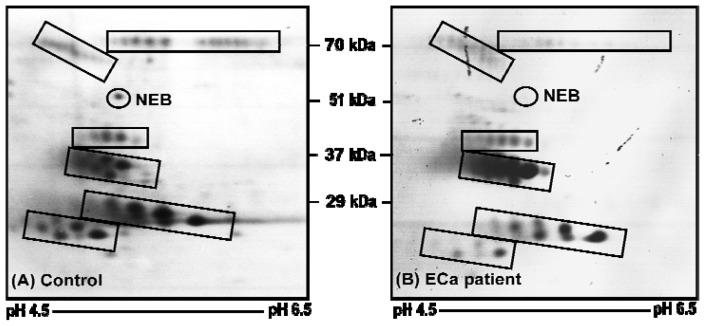
Typical 2-DE urinary *O*-glycoprotein profiles. Panels (**A**) and (**B**) refer to 2-DE urinary *O*-glycoprotein profiles of a control subject and a patient with stage IB ECa, respectively. Spot clusters that are marked in boxes are proteins, which were consistently detected by CGB lectin. NEB refers to nebulin. The acidic side of the membranes is to the left and the relative molecular mass declines from the top.

To exclusively target the *O*-glycoproteins in the urine samples, 2-DE separated urinary proteins were transferred onto a nitrocellulose membrane and probed with enzyme-conjugated CGB lectin. The 2-DE membrane profiles that were developed using the CGB lectin were entirely different from those generated by silver staining (Figure 2). Six clusters of *O*-glycoproteins were consistently detected in the urinary membrane profiles. In addition, one *O*-glycoprotein spot, with a molecular mass of approximately 51 kDa, seemed to appear in the profiles of all the control samples that were analyzed but faintly appeared in only one of the urine samples from patients with ECa. When the membrane profiles were subjected to image analysis, the levels of all urinary *O*-glycoproteins appeared comparable between the ECa patients and the controls except for the 51 kDa protein spot, which was 10.6-fold higher in the controls compared to the ECa patients (Table 3, panel A). To identify the sole aberrantly expressed urinary *O*-glycoprotein, the spot was excised and subjected to on-membrane trypsin digestion and analyzed by mass-spectrometry. Panel B of Table 3 demonstrates the MS/MS data obtained, which identified the *O*-glycoprotein spot as that of nebulin, when a database search was performed.

### 2.4. Immobilized CGB Lectin Affinity Chromatography and LC-MS/MS

To validate the data that was obtained from the Western blot-lectin analysis, pooled urinary protein samples from the ECa patients as well as those of the controls were independently subjected to CGB lectin affinity chromatography and their bound fractions were then analyzed using LC-MS/MS. Based on a minimum of 90% protein probability confidence, more than 200 peptides were considered identified, which included that of nebulin. Table 4 lists the *O*-glycosylated proteins that were isolated using the CGB lectin or those potentially known to be *O*-glycosylated. The nebulin peptide spectrum with the de novo sequence K.AYELQSDNVYKADLEWLRGIGWMPNDSVSVNHA.K (amino acid positions 4103–4136) apparently appeared only in the profile that was generated from the pooled control urine samples but not in that which was obtained from the pooled urine of ECa patients.

## 3. Discussion

In the present study, the different altered levels of ZAG, AAG and CD59 were initially detected in the urine samples of patients with ECa compared to the controls. While the levels of ZAG and AAG were significantly increased, the expression of CD59 was much lower in the ECa patients. These proteins have been previously identified as potential biomarkers for various different types of cancers (Table 5).

ZAG generally functions as a stimulator of lipolysis although it is also known to have other functions such as being a carrier protein, an immunoregulator and a cell adhesion molecule [15]. At the same time, ZAG has also been associated with cancer, as the levels of the protein have been reported to be increased in the sera of patients with cancers of the prostate [16] and cervix [7]. This was postulated to be due to the changes in the levels of adipokines and estrogen in the patients. The increase in levels of ZAG in the urine of patients with ECa shown in this study may reflect the change that occurred in the serum.

A comparable trend of changes in both the serum and urine samples was also observed in the case of AAG in patients with ECa. AAG was also found to be elevated in the urine of ECa patients in this study. And other researchers have previously reported its increased levels in the serum of patients suffering from several different cancers including ECa [26,29].

The decrease in the levels of CD59 in the urine of patients with bladder cancer [4], pancreatic ductal adenocarcinoma [30] and ovarian cancer [5] has been previously reported. CD59, also known as protectin, is a cell surface molecule that functions to inhibit the membrane attack complex of the complement pathway. The data of our present study further demonstrates decreased levels of CD59 in the urine of patients with ECa. Our data is also compatible with the report on the altered expression of CD59 in malignant tissues obtained from patients with ECa [19].

When similar 2-DE separated urinary proteins were transferred onto nitrocellulose membranes and probed with enzyme-conjugated CGB lectin, a different profile consisting of only *O*-glycosylated proteins was obtained. Comparative densitometry analysis of the 2-DE membrane profiles generated from patients and controls demonstrated the significant altered levels of a single *O*-glycoprotein, which was subsequently identified as a 51 kDa fragment of nebulin. The nebulin fragment spot may not be detected in the earlier 2-DE experiments probably because of its relatively low amount in the urine samples. The absence or low levels of the nebulin fragment in the urine of patients with ECa relative to the controls was further confirmed when urinary *O*-glycosylated protein fractions obtained from passing pooled urine samples to CGB lectin affinity chromatography were analyzed using LC-MS/MS.

Nebulin, a protein with a deduced molecular weight of 772.9 kDa functions as a template for polymerization of actin [31]. The protein is expressed predominantly in the thin filaments of striated muscle. It is known to be glycosylated, although the precise structure of its glycan moiety has never been characterized [32]. Hence, the result of the CGB lectin analyses performed in this study is a form of evidence that nebulin is *O*-glycosylated. The low molecular weight of the nebulin spot detected in the 2-DE experiments is indicative of a truncated or cleaved protein. The reduced levels of tissue nebulin are commonly associated with myopathy [33]. However, its increased levels in the pancreatic juice are believed to be associated with the cellular turnover in patients with pancreatic cancer [27]. To the best of our knowledge, there is no previous report on the detection of nebulin or its fragment in the urine, but the protein has been detected in other biofluids such as serum [34] and pancreatic juice [27].

## 4. Experimental Section

### 4.1. Urine Samples and Processing

Urine samples were obtained from patients newly diagnosed with stages IB and IIA/B ECa (*n* = 7) in the morning at the Gynecological Clinic, University of Malaya Medical Centre (UMMC), Kuala Lumpur, prior to any treatment or surgery. All patients were confirmed with negative diagnosis for other diseases. Table 6 demonstrates the clinical data of patients involved in the study. Control urine samples (*n* = 11) were obtained randomly from age-matched healthy women. Samples were obtained with the patients’ consent and approval granted by the Ethical Committee of UMMC in accordance to the ICH GCP guideline and the declaration of Helsinki. The samples were immediately added with sodium azide to a final concentration of 20 mM to inhibit any bacterial growth. To remove cells and debris, the samples were centrifuged at 10,000 rpm at 4 °C. The supernatant was collected and dialyzed against four changes of 1 L unsterile distilled water at the same temperature in order to reduce the concentration of salt, which would affect the subsequent analyses. The urine samples were aliquoted, freeze-dried and kept at −80 °C for a period of not more than a month. The protein content was determined using the Pierce BCA protein assay kit (Thermo Fisher Scientific, Rockford, USA).

### 4.2. Two-Dimensional Electrophoresis and Silver Staining

2-DE was performed as previously reported [35,36]. Briefly, 300 μg of freeze dried urine samples were solubilized and subjected to isoelectric focusing in 11 cm rehydrated precast Immobiline Drystrips pH 3–10 overnight, followed by first dimension separation with IPGphor 3 (GE Healthcare Biosciences, Uppsala, Sweden). For the second dimension separation, the focused samples in the strips were subjected to electrophoresis using 12.5% polyacrylamide gel in the presence of SDS. Silver staining of the 2-DE gels was performed based on the method described by Heukeshorven and Dernick [37]. For mass spectrometry analysis, a modified silver staining method by Shevchenko *et al.*, was used [38].

### 4.3. Image Analysis

The silver stained 2-DE gels (as well as lectin-developed membranes in section 4.6) were scanned using ImageScanner III (GE Healthcare Bioscience, Uppsala, Sweden). Image analysis was restricted to protein spot clusters that appeared consistently within each group of urine samples. The levels of proteins in each sample were calculated as a percentage of volume contribution (% vol) in which the volume of contribution refers to the volume percentage of a protein taken against the total spot volume of all the proteins, in order to eliminate the possible variations due to staining. All values are presented as mean ± S.E.M (standard error of the mean). The Student’s t-test was used to analyze the significance of difference between controls and ECa patients. A value of *p* < 0.05 was initially accepted as significantly different. The false discovery rate control was then performed using the method of Benjamini and Hochberg [39]. The *p* value was then corrected, with those <0.0214 considered to be significantly different.

### 4.4. On-Membrane Digestion

On-membrane digestion was performed according to the method described by Luque-Garcia *et al*. [40], with modifications. Briefly, the nitrocellulose membrane was blocked with 1% PVP-40 (w/v) in TBS, washed three times with the same buffer and incubated with the HRP-conjugated CGB lectin. After the development of the membrane as previously described, the protein spot was excised and destained with 0.1% EDTA dihydrate for 30 minutes. Horseradish peroxidase-conjugated CGB lectin was stripped by washing the spot with 0.5 and 1.0 M melibiose in Tris-HCl pH 7.5 for 30 minutes each. Then, the stripped spot was blocked with 0.5% (w/v) PVP-40 in 100 mM acetic acid at 37 °C for 30 minutes and digested with 12.5 ng/μL trypsin in 50 mM NH_4_HCO_3_ buffer overnight. After digestion, the sample was dissolved in acetone, vortexed and incubated for 30 minutes. The peptide was dissolved in 10 mg/mL of CHCA in 1% TFA of 50:50 ACN and MilliQ water after the removal of acetone, which contained a nitrocellulose membrane.

### 4.5. MALDI-ToF Mass Spectrometry

Plugs of proteins of interest from the 2-DE gels were subjected to in-gel digestion as previously described [41]. Identification of a protein by MALDI mass spectrometry was performed on one of the representative gels, using the Applied Biosystem 4800 Proteomics Analyzer. The mass standard kit (Applied Biosystems/MDS Sciex, Toronto, Canada) was used as the calibrator for the MS/MS analysis. The data from the MS/MS was submitted to the MASCOT search engine for protein identification.

### 4.6. Western Blotting and Detection by CGB Lectin

CGB lectin was purified from the crude extracts of the champedak seeds using immobilized galactose affinity chromatography. The purity of the lectin was confirmed using SDS-PAGE 18% polyacrylamide gel. Western blotting was performed by electrophoretically transferring 2-DE-separated urinary proteins from gels onto nitrocellulose membrane (0.45 μm) using the NovaBlot Kit of Multiphor II Electrophoresis system (GE Healthcare Bioscience, Uppsala, Sweden) at 0.8 mA/cm^2^ for 1 hour. The membrane was blocked with 3% w/v gelatin in Tris-buffered saline Tween-20 (TBST), pH7.5 for 1 hour at room temperature and washed three times with the same buffer. Detection of transferred urinary *O*-glycoproteins was performed using horseradish peroxidase-conjugated CGB lectin. Blots were developed using metal enhanced DAB substrate kit for horseradish peroxidase (Pierce, Rockford, USA).

### 4.7. CGB Lectin Affinity Separation and LC-MS/MS

CGB lectin was conjugated to CNBr-activated Sepharose 4B (GE Healthcare Bioscience, Uppsala, Sweden), based on the manufacturer’s instructions. Column was equilibrated with PBS, pH 7.2, prior to the application of pooled urinary proteins (400 μg). Eluted fractions were monitored by measurement of absorbance at 280 nm. Elution of urinary *O*-glycoproteins was performed using 0.5 M of D-melibiose in PBS. Bound fractions collected from both columns were pooled, dialyzed and concentrated with the vivaspin column concentrator (Sartorius Stedim Biotech, Goettingen, Germany) and send to The Vincent Coates Foundation Mass Spectrometry Laboratory, Stanford University Mass Spectrometry for LC-MS/MS analysis.

## 5. Conclusions

In summary, the data of this study is suggestive of the potential use of urinary ZAG, AAG, CD59 and a 51 kDa fragment of nebulin as complementary biomarkers for ECa. However, this requires further extensive validation in a study that has to be carried out on clinically representative populations. Such a study cannot be possibly performed using the present gel- and lectin-based proteomics analyses. Nevertheless, with the identification of the potential urinary biomarkers in the present study, validation is easily carried out in a large-scale investigation using assays like the ELISA.

## Figures and Tables

**Table 1:** Mass spectrometric identification of 2-DE separated urinary proteins.

Protein Entry Name +	Protein Name	Accession Number #	Nominal Mass (kDa)/p*I*	MOWSE Protein Score	Sequence Coverage (%)
**KNG**	Kininogen	P01042	71/6.34	68	7
**IGHG3**	Ig g3 chain C region	P01860	41/8.46	16	3
AAG	α1-acid glycoprotein	P19652	23/5.03	241	16
ZAG	Zinc α2 glycoprotein	P25311	33/5.57	134	20
AMBP	Protein AMBP	P02760	39/5.95	50	12
IGKC	Ig l1 chain C region	P01834	11/5.58	141	32
CD59	CD59 glycoprotein	P13987	14/6.02	121	18

+ Protein entry names are from the UniProtKB database [12].

# Accession numbers are from the Mascot search engine [13].

**Table 2:** Mean percentage of volume contribution of urinary proteins.

Urinary Proteins	Mean % Vol ± SEM		*p*	Fold Changes *

	Control (*n* = 11)	ECa (*n* = 7)		
AAG	0.161 ± 0.072	2.746 ± 0.717	0.001	+17.1
ZAG	0.175 ± 0.045	2.184 ± 0.592	0.001	+12.5
CD59	2.575 ± 0.497	0.177 ± 0.070	0.002	−14.6
KNG	5.137 ± 1.826	0.945 ± 0.491	0.108	ns
IGKC	14.785 ± 2.197	18.840 ± 2.651	0.286	ns
AMBP	8.941 ± 1.706	12.837 ± 3.430	0.306	ns
IGHG3	4.014 ± 1.221	4.554 ± 1.706	0.807	ns

* Fold expression changes are relative to the control values; (+) increase in expression; (−) decrease in expression; ns – not statistically significant; A *p-*value of less than 0.0214 was considered significant.

**Table 3:** Relative expression (A) and identification of nebulin (B).

(A)	Mean % Vol ± SEM		*p*	Fold Changes
			
	Control (*n* = 11)	ECa (*n* = 7)		
	
	1.08 ± 0.172	0.102 ± 0.095	0.001	−10.6

**(B)**	**Accession Number ^#^**	**Nominal Mass (kDa)/p*I***	**MOWSE Protein Score**	**Sequence Coverage (%)**
	
	P20929	775/9.11	64	1 *

* relative to native nebulin molecule.

**Table 4:** List of *O*-glycosylated/potentially *O*-glycosylated proteins isolated using CGB lectin.

Protein Name	Accession Number	Subcellular Location	Glycan #
Protein AMBP	P02760	Secreted	*O*-linked
ATP synthase subunit beta, mitochondrial	P24539	Membrane	Potential *O*-linked
Serotransferrin	P02787	Secreted	*O*-linked
Transmembrane protein 110	Q86TLZ	Membrane	Potential *O*-linked
Phosphoinositide-3 kinase interacting protein 1	Q96FE7	Membrane	*O*-linked
Ribonuclease pancreatic	P07998	Secreted	Potential *O*-linked
Prostaglandin-H2-D isomerase	P41222	Secreted	Potential *O*-linked
Membrane bound transcription factor site-1 protease	Q14703	Membrane	Potential *O*-linked
Homeobox protein engrailed 2	P19622	Nucleus	Potential *O*-linked
Actin, cytoplasmic 1	P60709	Cytoplasmic	Potential *O*-linked
Neurofilament medium polypeptide	P07197	Cytoplasmic	*O*-linked
CD55 decay-accelerating factor splicing variant 4	Q14UF3	Membrane	Potential *O*-linked
Leucine rich alpha 2 glycoprotein	P02750	Secreted	*O*-linked
Spondin-2	Q9BUD6	Secreted	Potential *O*-linked
Protein RRP5 homolog	Q14690	Nucleus	Potential *O*-linked
CD44 antigen	P16070	Membrane	*O*-linked
Phosphatidylinositol-3,4,5-triphosphate 5-phosphatase-1	Q92835	Membrane	Potential *O*-linked
Solute carrier family 12 member 6	Q9UHW9	Membrane	Potential *O*-linked
Mucin-5B	Q9HC84	Secreted	*O*-linked
Major facilitator superfamily domain containing protein 10	Q14728	Membrane	Potential *O*-linked
PDZ and LIM domain protein 5	Q96HC4	Membrane	Potential *O*-linked
Leukocyte associated immunoglobulin-like receptor 1	Q6GTX8	Membrane	Potential *O*-linked
Tumor necrosis receptor superfamily member 16	P08138	Membrane	Potential *O*-linked
Lipocalin-1	P31025	Secreted	Potential *O*-linked
Amyloid beta A4	P05067	Membrane	*O*-linked
Nebulin	P20929	Cytoplasmic	Potential *O*-linked
Transcription factor HES-2	Q9Y543	Nucleus	Potential *O*-linked
Rho GTPase-activating protein 12	P20936	Cytoplasmic	Potential *O*-linked
Solute carrier family 13 member 3	Q8WWT9	Membrane	Potential *O*-linked
Sodium/bile acid contransporter	Q14973	Membrane	Potential *O*-linked
Bromodomain-containing protein 3	Q15059	Nucleus	Potential *O*-linked
Inter alpha trypsin inhibitor heavy chain H4	Q14624	Secreted	*O*-linked
Ig gamma-1 chain C region	P01857	Secreted	Potential *O*-linked
Ig gamma-2 chain C region	P01859	Secreted	Potential *O*-linked
Ig alpha-1 chain C region	P01876	Unknown	*O*-linked
Ig lambda-1 chain C region	P0CG04	Unknown	Potential *O*-linked

# Types of glycan as annotated by UniProt; glycosylation potential was determined by NetOGlyc 3.1 [14].

**Table 5:** Identification of CD59, ZAG, AAG and NEB as potential biomarkers. Check this table.

Protein	Type of Cancer	Sample Type	Reference
CD59	Oral	Saliva	[17]
	Lung	Urine	[18]
	Ovarian	Urine	[5]
	Endometrial	Tissue	[19]

ZAG	Breast	Tissue	[20]
	Hepatocellular	Tissue	[21]
	Prostate	Tissue and serum	[16]
	Cervical	Serum	[7]

AAG	Breast	Nipple aspirate fluid	[22]
	Colon	Plasma	[23]
	Hepatocellular	Serum	[24]
	Ovarian	Serum	[25]
	Endometrial	Serum	[26]

NEB	Pancreatic	Pancreatic juice	[27]
	Gastric	Tissue	[28]

**Table 6:** Clinical data of ECa patients involved in the study.

ECa Patient	Stage	Estrogen or Tamoxifen Treatment	Obesity	Late Menopause	Previous Pregnancy	Early Puberty	Blood Creatinine Level
1	IB	No	No	Yes	Yes	No	Normal
2	IB	No	No	Yes	Yes	No	Normal
3	IB	No	No	No	Yes	No	Normal
4	IB	No	No	No	Yes	No	Normal
5	IB	No	No	No	Yes	No	Normal
6	IIA	No	Yes	No	Yes	No	Normal
7	IIB	No	No	No	No	No	Normal
